# Apocrine carcinoma of the male breast: a case report of an exceptional tumor

**DOI:** 10.11604/pamj.2014.19.294.2973

**Published:** 2014-11-17

**Authors:** Mohammed Sekal, Kaoutar Znati, Taoufiq Harmouch, Afaf Amarti Riffi

**Affiliations:** 1 Department of Pathology, University Hospital Hassan II of Fez, Morocco

**Keywords:** Apocrine carcinoma, breast, man

## Abstract

Apocrine carcinoma of the male breast is an exceptional malignant tumor. It does not have a particular clinical or radiological appearance, but it's microscopically characterized by the presence of granular cells and foamy cells representing over 90% of tumor cells. These cells express most of the time the GCDFP-15 and the androgen receptors. This tumor is a distinct molecular entity. In this observation, we report the case of a 70 year old man presenting apocrine carcinoma of the left breast diagnosed at the stage of lung metastasis.

## Introduction

Apocrine carcinoma of the breast is a malignant tumor, microscopically characterized by the presence of granular cells and foamy cells representing over 90% of tumor cells. It does not have a particular clinical or radiological appearance, but it's a distinct molecular entity. We report an exceptional observation of apocrine carcinoma of breast in a man.

## Patient and observation

**Figure 1:**
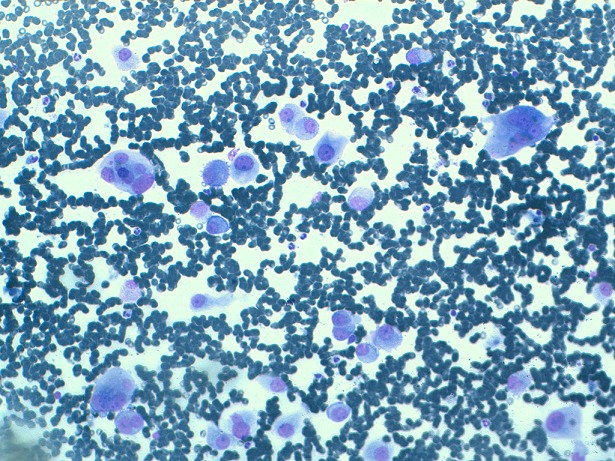
MGG stained cytology after a fine needle aspiration showing isolated apocrine cells with a large irregular nucleus, and abundant cytoplasm. The background is hemorrhagic

**Figure 2:**
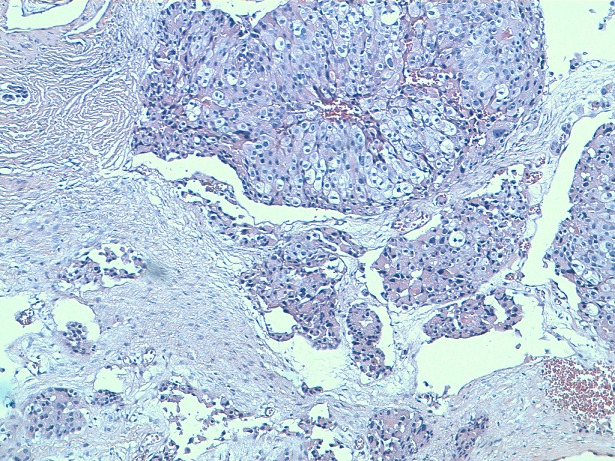
Carcinomatous proliferation forming clusters of cells with a large irregular nucleus, a prominent nucleoli and clear cytoplasmic borders (HES x100)

**Figure 3:**
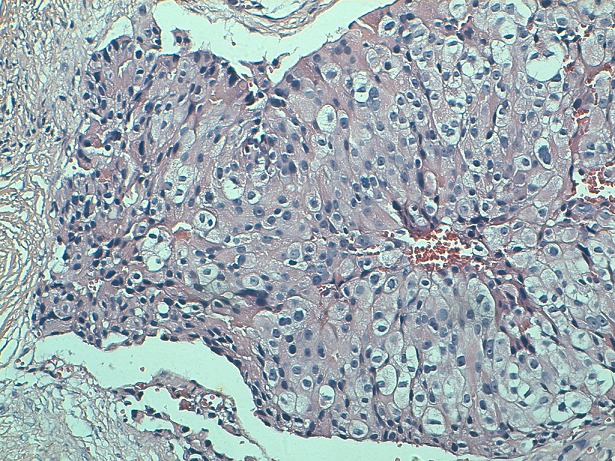
Type A cells are distinguished with abundant granular eosinophilic cytoplasm and type B cells with a micro-vacuolar cytoplasm (HES x200)

**Figure 4:**
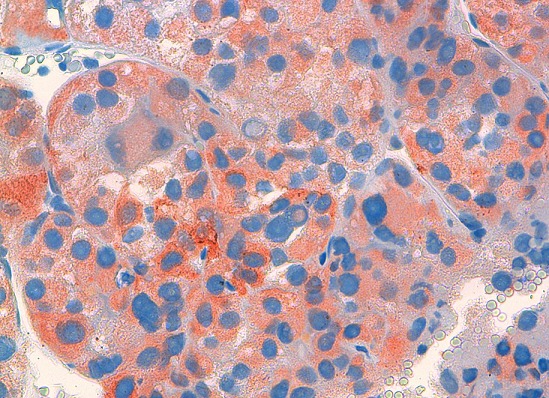
Immunohistochemical study shows a GCDFP-15 cytoplasmic expression in tumor cells

He's a 70 years old man who had for 9 years ago a painless nodule at the left breast. It was movable relative to superficial and deep plans. Then, this nodule presented a rapid augmentation of its volume with adhesion to both superficial and deep plans, and inflammatory opposite signs. Mammography showed an ACR5 lesion measuring 4 cm long axis. We realized a fine-needle aspiration cytology, a spread on slides and stained with May-Grünwald Giemsa (MGG) which showed isolated carcinomatous cells, sometimes forming three-dimensional clusters. Their nucleus is large and irregular with nucleolus. The cytoplasm is abundant. We did not observe myoepithelial cells and the background is necrotic and hemorrhagic (Figure 1). A biopsy of the tumor was performed. It objectified after Hematoxylin Eosin saffron Staining (HES) staining, a carcinomatous proliferation forming clusters and cellular cords. The tumor cells showed a large and irregular nucleus with prominent nucleoli and abundant cytoplasm, sometimes eosinophilic and granular, and sometimes micro-vesicular. Cytoplasmic borders are clear (Figure 2, Figure 3). The number of mitoses was estimated at 7 per 10 fields. An immunohistochemical study showed that tumor cells express GCDFP-15 (Figure 4), but do not express estrogenic and progesterone receptors, nor Her 2. The diagnosis of apocrine carcinoma of the breast is made. The staging has objectified the presence of lung metastases. The patient received palliative chemotherapy. He died after 8 months of evolution.

## Discussion

Apocrine carcinoma of the breast is a rare malignant tumor whose incidence varies between 0.3% and 4% of all female's breast cancer [1]. This tumor is exceptional in men. Indeed, only a dozen cases have been described in the literature [2]. Like our patient, the average age of onset is between the sixth and seventh decade [1]. Its clinical mammographic and sonographic characteristics mimic those of non-apocrine breast invasive carcinoma. The cytology after fine needle aspiration may have a role in orienting. As in our patient, it shows apocrine cells with cell and nuclear atypias, a large irregular nucleus and a prominent nucleolus with abundant cytoplasm [3]. Macroscopically, apocrine carcinoma is in the form of a nodule or endo-cystic growths, often multicentric [1].

Histologically, the tumor is defined as a carcinoma showing in more than 90% of tumor cells, cytological features of apocrine cells (mixture of varying proportions of type A cells and type B cells). Type A cells have abundant cytoplasm containing eosinophilic, PAS positive, diastase-resistant granular. This is due to an abundance of large mitochondria, some of which have abnormal peaks. Type B cells have a micro-vacuolar cytoplasm resembling foamy histiocytes or sebaceous cells. The tumor cells have visible cytoplasmic borders. Some apocrine carcinomas are exclusively composed of type A cells. In this case, the differential diagnosis is granular cell tumors. Other apocrine carcinomas are composed exclusively of type B cells. In this case, the differential diagnosis is histiocytic proliferation and inflammatory reactions. The distinction is made by immunohistochemical study showing positive staining of apocrine carcinoma by anti-cytokeratin antibodies [1].

Although apocrine carcinoma is a distinct histological entity, however, there is no sensitive and specific immunohistochemical marker for confirming apocrine differentiation. Immunohistochemical study shows an expression of GCDFP-15 in 76% to 100% of cases. The GCDFP-15 is a glycoprotein originally isolated from breast cyst fluid. It's localized in cytoplasmic vesicles, and in osmiophilic granules. With the development of this marker, a more objective diagnostic criterion has been introduced [4]. Androgen receptors are expressed in 54% of cases [5]. Moreover, tumor cells can express B72.3, estrogenic-beta receptors, HER2, p53 and Ki-67 [1]. Usually, these tumors do not express the estrogen receptor-alpha, progesterone receptors and bcl-2. [1] Concerning the molecular study, we note the presence of abnormalities in the long arm of chromosome 7 (genes encoding the GCDFP-15 and prolactin-inducible protein), also the loss of heterozygosity for the TP53 gene, the VHL gene (3p25), the NB gene (1p35-36), and PKD1/TSC2 gene (16p13) [6]. The new molecular classification of breast cancers based on studies of CGH-array, classifies apocrine carcinoma individually [7].

Treatment protocols are similar to those of non-apocrine carcinoma. However, studies involving the use of anti-androgens are in progress [6]. The survival rate at 5 years was significantly better for the apocrine carcinoma (72%) with a longer time to recurrence compared to non-apocrine carcinoma. Prognostic factors are essentially the mitotic account and TNM stage. [1]

## Conclusion

Apocrine carcinoma of the breast is a distinct histological and molecular entity. It is rare in women and exceptional in man. Studies are being conducted to use anti-androgens such as targeted therapy.
